# *Helicobacter* spp. in the Stomach of Cats: Successful Colonization and Absence of Relevant Histopathological Alterations Reveals High Adaptation to the Host Gastric Niche

**DOI:** 10.3390/vetsci9050228

**Published:** 2022-05-10

**Authors:** Sílvia Teixeira, Dulce Filipe, Manuela Cerqueira, Patrícia Barradas, Francisco Cortez Nunes, Fátima Faria, Freddy Haesebrouck, João R. Mesquita, Fátima Gärtner, Irina Amorim

**Affiliations:** 1 ICBAS—School of Medicine and Biomedical Sciences, University of Porto, 4050-313 Porto, Portugal; silvia.goncalves.teixeira@gmail.com (S.T.); franciscojvcnunes@gmail.com (F.C.N.); fatimafaria10@yahoo.com.br (F.F.); fgartner@ipatimup.pt (F.G.); 2 Institute for Research and Innovation in Health (i3S), University of Porto, 4200-135 Porto, Portugal; 3 Institute of Molecular Pathology and Immunology of the University of Porto (IPATIMUP), 4200-135 Porto, Portugal; 4 VetFilipe, 4740-674 Barcelos, Portugal; dulcef@live.com.pt; 5 Polytechnic Institute of Viana do Castelo (IPVC), Agrarian School, 4990-706 Ponte de Lima, Portugal; manuelacerqueira@esa.ipvc.pt; 6 Epidemiology Research Unit (EPIUnit), Institute of Public Health, University of Porto, 4050-091 Porto, Portugal; patricia.barradas@ispup.up.pt (P.B.); jrmesquita@icbas.up.pt (J.R.M.); 7 University Institute of Health Sciences, Advanced Polytechnic and University Cooperative (CESPU), 4585-116 Gandra, Portugal; 8 Department of Pathobiology, Pharmacology and Zoological Medicine, Faculty of Veterinary Medicine, Ghent University, B9820 Ghent, Belgium; freddy.haesebrouck@ugent.be; 9 Department of Veterinary Clinics, School of Medicine and Biomedical Sciences (ICBAS), University of Porto, 4050-313 Porto, Portugal

**Keywords:** feline gastric mucosa, cats, non-*Helicobacter Pylori Helicobacters* (NHPH), *Helicobacter pylori* (HP), histochemistry, immunohistochemistry (IHC), polymerase chain reaction (PCR), stomach

## Abstract

In addition to *Helicobacter pylori*, many non-*Helicobacter pylori* Helicobacters (NHPH) are able to cause gastric disease in humans. Cats are a natural reservoir for many of these species. Accordingly, living in close and intimate contact with animals has been identified as a risk factor, and an important zoonotic significance has therefore been attributed to NHPH. To determine the prevalence and associated gastric histopathological changes of *Helicobacter* species, the gastric mucosa of 71 cats were evaluated. Only four presented normal histopathological mucosa with the absence of spiral-shaped organisms. Normal gastric mucosa and the presence of spiral-shaped bacteria were observed in 13 cats. The remaining animals presented histopathological changes representative of gastritis. *Helicobacter* species were detected in 53 cats (74.6%) by at least one detection method. None of the animals were positive for *H. pylori* or for *H. ailurogastricus*. *Helicobacter heilmannii* organisms were identified in 20 animals, predominantly in the body gastric region. *Helicobacter salomonis* was the second most prevalent species (57.1%), although it was mainly found in association with other NHPH. *Helicobacter felis* and *H. bizzozeronii* were less frequently detected. The great majority of the *Helicobacter* spp. PCR-positive animals presented normal features regarding fibrosis/mucosal atrophy, neutrophils, eosinophils, or other inflammatory cells and lymphofollicular hyperplasia. Given the controversy and the strong evidence of absence of significant histopathological alterations associated with the presence of *Helicobacter* spp. in cats, it is possible to hypothesize that these bacteria may be able to adapt to the feline gastric microenvironment or even to comprise part of the gastric microbiome of this animal species. Thus, prudency must be taken when prescribing an antibiotic therapy based solely on the presence of these bacteria in the feline stomach.

## 1. Introduction

The genus *Helicobacter* is currently composed of about 48 validated species [1]. *Helicobacter pylori* (HP) is the most common and known species in men, but other distinct *Helicobacter* species have also been observed in the stomachs of humans and several animals [2,3,4]. The group of non-*Helicobacter pylori Helicobacters* (NHPH) that mainly colonizes the gastric mucosa of cats and dogs includes *H. felis*, *H. bizzozeronii*, *H. salomonis*, *H. cynogastricus*, *H. baculiformis,* and *H. heilmannii* sensu stricto [3]. Most of these NHPH can trigger gastric pathologies in humans such as gastritis, gastric ulceration, and gastric mucosa-associated lymphoid tissue (MALT) lymphoma [3]. NHPH most likely originate from animals, being found in about 0.2–6% of human gastric biopsies, thus further underlining the relevance of these bacteria as zoonotic agents [3,5,6].

On the other hand, NHPH are commonly found in the stomachs of healthy cats (42–100%) or in animals presenting clinical signs of gastrointestinal disorders, such as chronic vomiting and diarrhea (53–76%) [7,8,9,10,11]. Nevertheless, the pathogenicity of *Helicobacter* spp. is not clearly understood or recognized in pets [3,9].

Epidemiological and clinical data regarding NHPH in feline stomachs are variable, but only few studies have determined the specific species present in these hosts [12,13]. According to the literature, the predominant feline gastric *Helicobacter* species are *H. felis*, *H. bizzozeronii* and *H. heilmannii*, and *H. salomonis* is less often detected [3,10,12,14]. Although its prevalence has not yet been established, *H. baculiformis* was also isolated from the stomach of a cat [15]. Additionally, the presence of *H. pylori* in feline stomachs has been seldom reported [16,17,18,19], and this finding brought attention to the putative role of cats as reservoirs [18,19]. However, the distribution and incidence of the various *Helicobacter* species in cats appears to be influenced by local geography and climate [20].

Some investigations have reported the preference of *Helicobacter* species to colonize the oxyntic gastric mucosa [12,19,21,22], but the great majority have not found significant differences regarding NHPH colonization density between the various compartments of the feline stomach [9,18,23,24,25,26]. Nevertheless, mixed infections involving different *Helicobacter* species commonly occur in cats [3,10,17,18,23].

A lack of knowledge about the pathogenicity of *Helicobacter* infections in both dogs and cats makes treatment decisions difficult. Furthermore, in many veterinary studies, *Helicobacter* infections have been difficult to eradicate [27,28]. Although many dogs and cats treated with the pharmacological combinations classically used in human medicine did not experience long-term eradication when re-tested, in many of these patients, the frequency of vomiting and the gastric lesion severity improved with therapy. Furthermore, it has been shown that acquired antibiotic resistance often occurs in bacteria associated with dogs and cats [29,30]. Therefore, from a public health perspective and always considering animal health and welfare promotion, the choice of treatment must be judicious. 

In the present study, histological, immunohistochemical, and molecular diagnostic techniques were used in order to assess the occurrence of *Helicobacter* spp. in distinct regions of the feline stomach and to correlate it with putative histopathological alterations.

## 2. Materials and Methods

### 2.1. Sample Collection

Gastric tissues were obtained from 71 cats (36 females, 28 males, and 7 without sex information) ranging from 6 months to 16 years old. The samples were randomly selected from the archives of the Laboratory of Veterinary Pathology, ICBAS-UP (Portugal), where they were received between 2009 and 2019. Samples were collected from 20 cats during endoscopic procedures, from 4 during surgery, and from 47 during necropsy examinations. All these procedures were performed in a clinical context while attempting to treat the animals based on the best clinical judgment of their attending practitioners. None of the actions were taken solely for research purposes, and the researchers had no influence on the selections and execution of such procedures. 

All gastric samples were collected in duplicate: one was used for histopathological analysis, and the other was used for molecular procedures. For histological and immunohistochemical purposes, tissues were preserved in 10% neutral buffered formalin and embedded in paraffin wax. Three 3-µm-thick serial sections of were made, one being stained with hematoxylin–eosin (HE), another being stained with modified Giemsa (MG) stain, and the third being used for immunohistochemistry (IHC). For the molecular investigation, the samples were kept at −20 °C until DNA extraction.

### 2.2. Histological Evaluation

Two observers independently assessed gastric samples (IA and FG). The World Small Animal Veterinary Association (WSAVA) guidelines were adopted to evaluate histopathological parameters including alterations in cellularity, lamina propria fibrosis, and gland atrophy [31]. The degree of morphological features and inflammatory changes was graded as normal, mild, moderate, or marked using the available WSAVA gastrointestinal standardization visual analogue [31].

A microscopic examination of the complete section of the gastric tissue was carried out. HE and MG histological stains and IHC were used to determine the presence of *Helicobacter* spp. When one of these methods yielded a positive result, a cat was considered as *Helicobacter*-positive. Additionally, bacterial density colonization was quantified: +, few organisms (<10 organisms/400×); ++ moderate number of organisms (from 10 to 50 organisms/400×); and +++ large number of organisms (>50 organisms/400×) [4].

### 2.3. Immunohistochemistry

Sections were deparaffinized in xylene and rehydrated in sequential graded alcohols, and antigen retrieval was accomplished in a water bath in a 10 mmol/L sodium citrate buffer at pH 6.0 for 20 minutes (min). Slides were chilled for 10 min at room temperature and washed twice for 5 min in triphosphate buffered saline (TBS). For visualization, the NovolinkTM Max-Polymer detection system (Novocastra^®^, Newcastle, UK) was used, following the manufacturer’s instructions. Sections were incubated overnight at 4 °C, with a polyclonal anti-serum against *H. pylori* (RBK012; Zytomed, Berlin, German) (1:200) that has been shown to have immunoreactivity with a wide assortment of *Helicobacter* genus bacteria [4]. Sections were rinsed with TBS between each step of the procedure. Color was developed with 3,3′-diamino-benzidine (DAB) (Sigma^®^, St. Louis, MO, USA), and sections were then lightly counterstained with hematoxylin, dehydrated, and mounted. Negative controls were performed by replacing the primary antibody with an antibody of the same immunoglobulin isotype at the same original concentration. Positive immunoreactivity was recorded as a distinct golden-brown labelling of the bacteria located on the mucosal surface, in gastric pits or glands, and inside parietal cells. 

### 2.4. DNA Extraction, PCR Amplification and DNA Sequencing

DNA was extracted from gastric frozen tissue samples using the EXTRACTME^®^ DNA tissue kit (BLIRT, Gdańsk, Poland), according to the supplier’s instructions. The first molecular approach was a *Helicobacter* genus-specific PCR assay. All samples that were shown to be positive for the *Helicobacter* genus were subjected to a *Helicobacter* species-specific conventional PCR assay for *H. pylori*, *H. heilmannii*, *H. felis*, *H. bizzozeronii*, *H. salomonis,* and *H.* ailurogastricus following previously described protocols (Table S1) [4,32].

Aliquots of each PCR product were electrophoresed on 1.5% agarose gel and analyzed under UV light for the presence of specific fragments using Xpert Green Safe gel stain (GRISP, Porto, Portugal). DNA fragment size was compared with the standard molecular weight via a 100 bp DNA ladder (GRISP, Porto, Portugal) and the molecular weight of the positive controls (Table S1). Positive controls were DNA-isolated from pure cultures of each tested *Helicobacter* species (Table S1), whereas negative controls were DNA-isolated from distilled water.

To rule out false-positive results, each positive sample’s amplicons were subjected to bidirectional sequencing using the Sanger method at the IPATIMUP’s Genomics core facility, Porto, Portugal. BioEdit^®^ Sequence Alignment Editor version 7.2.5 was used for sequence editing and multiple alignments. Using the non-redundant nucleotide database (http://blast.ncbi.nlm.nih.gov/Blast.cgi, accessed on 1 October 2021), the sequences were subject to the basic local alignment search tool (BLAST) [33]. The DNA sequences were deposited in Genbank^®^.

### 2.5. Statistical Analysis

Cohen’s kappa coefficient was determined to evaluate the level of agreement or reproducibility between the methods used here for *Helicobacter* spp. diagnostics. The results were interpreted as previously described in the literature [34].

The chi-squared test was used to assess differences between gastric histopathological alterations and the presence of *Helicobacter* spp. Differences were considered statistically significant at of *p*-values of <0.05. Analyses were performed using GraphPad Prism 5 (GraphPad Software Inc., La Jolla, CA, USA).

## 3. Results

### 3.1. Animals and Samples

A total of 82 gastric samples (61 from the body region and 21 from the antrum region) originating from 71 cats were analyzed. In 16 of the 71 animals, both gastric regions were accessible for evaluation, whereas only body or antrum regions were available in 45 and 5 cats, respectively. The histopathological evaluation was not possible to perform in 5 of the 66 gastric body samples (Table 1).

### 3.2. Histological Evaluation of Feline Gastric Samples

Among the 66 cats subjected to histopathological evaluation, 7 presented normal gastric features with absence of spiral-shaped organisms (10.6% or 7/66). Normal gastric mucosa and the presence of NHPH were observed in 13 cats (19.7% or 13/66). The remaining animals presented histopathological changes compatible with gastritis (46/66 or 69.7%) (Table 2). Based on the observed histopathological alterations, both mild and moderate chronic gastritis were frequently diagnosed in 30.3% (20/66) and 31.8% (21/66), respectively, of the total animals. Marked chronic gastritis affected 7.6% (5/66) of the microscopically analyzed feline stomachs.

Epithelial injuries were observed in the surface and the gastric pits of both gastric regions. In the body region, in addition to mild-to-moderate epithelial injuries, we classified one case as marked in both the surface and the gastric pits. In the antrum region, we found marked epithelial injuries in the surface of 14.3% (3/21) samples, whereas in the gastric pits, it was observed only in one case.

Abnormal intraepithelial lymphocyte infiltration was observed in 30 animals (30/62), but this parameter was not possible to evaluate in 4 animals due artefactual constraints that resulted in a lack of proper epithelial representation (Table 2).

Mild-to-moderate gastric mucosal atrophy, glandular nesting, or fibrosis was visualized in 37.7% (23/66) of cats, being more pronounced in the antrum region of the feline stomach (Table 2).

Abnormal neutrophilic infiltration was observed in eight cats (8/66) (Table 2). Marked neutrophilic infiltration and extensive ulceration was observed in one cat (1/66). Other inflammatory cells, as signified by the mild infiltration of mast cells, were visualized in the body region of one animal and in the antrum of four animals.

### 3.3. Microscopic Helicobacter spp. Identification in Feline Gastric Samples

Of all the animals, 74.6% were positive (53/71) and 25.4% were negative (18/71) for *Helicobacter* spp. regardless of the test used to detect the bacteria. Overall and regardless of stomach location, *Helicobacter* spp. were observed using HE, MG, and IHC in 37.9%, 48.5%, and 71.2% of the cats, respectively (Table 3). 

As the method that presented the highest detection rate, immunohistochemistry allowed for the identification of high bacterial density (>50 organisms) in the great majority of cases. These were often observed in the superficial mucus, within the gastric glands, and in the cytoplasm of parietal cells.

Regarding the different gastric regions, with routine staining, spiral-shaped bacteria were detected in 34.4% of body samples and 47.6% of antral samples. Using MG stain, bacteria were found in 44.3% of body samples and 76.2% of antral samples. The *Helicobacter* antigen was detected with immunohistochemistry in 70.5% of the body samples and in 71.4% of the antral samples (Table 3). 

When evaluating the level of agreement between the three diagnostic methods and considering the robustness of IHC and the low rate of false-positive results provided by this technique [35], MG showed the best matches (% of agreement: 70.8%; Cohen’s k: 0.41), followed by HE and PCR (% of agreement: 63.1%; Cohen’s k: 0.33).

### 3.4. Helicobacter Species Identification through PCR Analysis

Further identification at the species level was performed using *Helicobacter* species-specific conventional PCR, which enabled NHPH detection in 39.4% of the animals (28/71) (Table 3). 

Amongst the 28 PCR-positive cases, 24 were also IHC-positive, one was IHC-negative, and three were not possible to immunohistochemically evaluate due to improper tissue preservation conditions.

In 32.1% of the positive samples (9/28), only one *Helicobacter* species was identified, and mixed infections were detected in 67.9% of the positive samples (19/28). 

*H. heilmannii* organisms were the most commonly found (71.4%), being identified in three cats as a single infection and in 17 cats as mixed infections. Additionally, in two cats, the obtained amplicons presented approximately 92% homology with *H. heilmannii*, so these cases were reclassified as *H. heilmannii*-like [4].

*H. salomonis* was the second most prevalent species (50%), although it was mainly found in association with other NHPH (85.7%) rather than alone (14.3%). Similarly, *H. felis* colonized 46.4% of the positive animals, also in association with other NHPH (92.3%) rather than alone (7.7%). The least detected species was *H. bizzozeronii,* identified in 39.3% of the cats (Table 4). None of the animals tested positively for *H. pylori* or *H. ailurogastricus*.

Mixed infections with *H. heilmannii* and *H. salomonis* were the most frequently encountered (17.9%). In the body area, the most identified species was *H. heilmannii* (10.7%) whereas in the antrum region, all infections were associated with more than one species (Table 4).

### 3.5. Presence of Helicobacter spp. and Histopathological Alterations in Feline Gastric Samples

The results of the histopathological alterations and colonization density observed in the feline stomach, according to the *Helicobacter* species-specific PCR results and the final *Helicobacter*-positive and-negative results regardless of the method used, are detailed in Table S2. 

Many animals positive for *Helicobacter* spp. did not show relevant histological alterations (24.5% or 13/53). Indeed, the great majority of the *Helicobacter* spp. PCR-positive animals presented normal features regarding fibrosis/mucosal atrophy, neutrophils, eosinophils, or other inflammatory cells and lymphofollicular hyperplasia (Table S2). 

Although not significantly, 31 of 53 (58.5%) *Helicobacter* spp. PCR-positive animals characteristically presented the mild-to-moderate infiltration of lymphocytes and plasma cells into the lamina propria. There were no statistically significant results regarding the occurrence of gastritis between *Helicobacter* PCR-positive and PCR-negative animals (67.9% vs. 55.8%, respectively) (Table S2). Additionally, although a higher density of colonization was observed, none of the cases diagnosed with multiple *Helicobacter* species elicited a pronounced inflammatory response. 

When evaluating the association between gastric histopathological alterations and the presence of *Helicobacter* spp., a statistically significant difference was only observed regarding intraepithelial lymphocyte infiltration: a significantly higher percentage of *Helicobacter*-positive samples presented abnormal intraepithelial lymphocyte infiltration (86.7% versus 62.5%; *p* = 0.029762) (Table 5).

## 4. Discussion

Most cats carry helicobacters in their gastric mucosa [3]. Spiral-shaped bacteria are commonly identified in the stomachs of cats both presenting signs of gastritis and considered clinically healthy. Therefore, the presence of these organisms in cats remains of uncertain clinical significance [3,12].

In the present study, the presence of *Helicobacter* spp. was assessed with four methods (HE, MG, IHC and conventional PCR). It should be noted that all these diagnostic methods have their own limitations and differ in terms of sensitivity and specificity. Thus, each technique should be used in very specific contexts and their combination should ideally be taken into account to obtain more reliable results. Accordingly, an overall *Helicobacter* spp. occurrence of 74.6% was found in this study. These high values are consistent with those available in the literature, with previous studies reporting the presence of these bacteria in 64% [21], 85% [36], 87% [25], 67.5% [13], and 94.6% [26] of animals tested.

Here, the most frequently detected *Helicobacter* species was *H. heilmannii* (71.4%), followed by *H. salomonis*. Kubota-Aizawa et al. [12] showed that half of the Japanese cats screened in their study were infected with *Helicobacter* species, with the most prevalent being *H. heilmannii* (89.3%), followed by *H. felis* and *H. bizzozeronii* (both at 25%). Similarly, in another publication, *H. heilmannii* was also the most identified species (17/20) and *H. felis* was only identified in coinfections (2/17) [25]. As previously reported, mixed infections with *Helicobacter* spp. are a common and interesting finding in dogs and cats [3,4,12,17,18]. In the present study, the mixed colonization of the same niche by two or more *Helicobacter* species was detected in 67.9% of the animals. Furthermore, the obtained *Helicobacter* spp.-specific PCR results highlighted the richness and diversity of the bacterial combinations that can be found in the feline stomach. 

It is worth mentioning that, although only affecting two cats, *H. salomonis* was identified here for the first time as a single agent, which is in contrast with most previous investigations in which it was mainly found in association with other species [10]. 

The prevalence and pathogenic value of the recently discovered *H. ailurogastricus* remains unknown [29]. To the best of our knowledge, this investigation is the first to assess the presence of *H. ailurogastricus*, a species closely related to *H. heilmannii* in the feline stomach. Nevertheless, this species was not identified in this group of cats.

Although *H. pylori* has occasionally been found in the stomach of cats, the importance of this animal species as a possible reservoir is probably low [16,17,18,19]. Additionally, both in the present study and previous ones, this species was not detected at all [12].

In this study, 69.7% of the animals presented histopathological changes compatible with gastritis. Amongst those diagnosed with *Helicobacter*, 67.9% also presented inflammation. However, amongst the group of *Helicobacter*-negative animals, 55.8% also displayed microscopic findings suggestive of gastritis. In other investigations and regardless of the presence of gastric *Helicobacter* spp., most of the cats also presented mild-to-moderate gastritis [26]. In addition, in another publication, no significant differences between *Helicobacter* spp.-positive and -negative cats and the severity of chronic gastritis were reported [12]. Again and with precedent, we failed to demonstrate a possible association between the presence of this bacterium and gastric inflammation. Furthermore, the presence of an inflammatory response may or may not be related to bacterial density. However, in this study, the great majority of the *Helicobacter*-positive cats were colonized with a large number of bacteria (68.5%). Additionally, although a higher density of colonization was observed, none of the cases diagnosed with multiple *Helicobacter* species elicited a pronounced inflammatory response.

In the present study, no evidence for an association between gastritis; gastric pit epithelial injury; fibrosis/mucosal atrophy; the lamina propria infiltration of eosinophils, neutrophils, or macrophages and gastric follicles; and the presence of *Helicobacter* was found. Indeed, only abnormal intraepithelial lymphocyte infiltration was significantly associated with the presence of *Helicobacter* organisms. The same association was previously found in dogs [4]. To date, it is not clear whether gastric intraepithelial lymphocytosis should be considered a *Helicobacter*-induced finding that could help in future diagnoses or whether it only reflects an unspecific underlying mechanism of the diffuse inflammation of the gastrointestinal tract with limited diagnostic relevance.

Given the controversy and the strong evidence of the absence of significant histopathological alterations associated with the presence of *Helicobacter* spp. in cats, it is possible to hypothesize that these bacteria may be able to adapt to the feline gastric microenvironment and possibly comprise part of the gastric microbiome of this animal species. Norris et al. (1999) already raised similar questions when they verified that the gastric mucosa of healthy pet cats were commonly colonized by *Helicobacter* species with no related signs of gastritis [9]. The absence of gastric disorders and the non-existence of a pronounced associated immunological response might be indicative of a highly effective regulatory process that protects against *Helicobacter* infection or eventually recognizes these bacteria as belonging to the host’s gastric microbiome. Already at that time, the authors suggested a probable commensal or perhaps a symbiotic relationship between *Helicobacter* spp. with their feline hosts [9,37]. 

Moreover, previously Jergens et al. (2009) conducted an investigation in which two cats were infected and treated with antibiotics and fed with an elimination diet for 21 days. Then, the *Helicobacter* status was evaluated and compared before and after antibiotic therapy by different methods. After treatment, HE and Warthin Starry stains failed to disclose spiral bacteria in tissues from any of the animals, FISH confirmed the clearance of *Helicobacter,* and PCR was negative. However, the severity of gastritis did not appreciably differ between biopsies obtained before and after triple drug therapy [38]. 

Due to the extensively applied broad-spectrum antimicrobial agents and their close contact with humans, companion animals are assumed to be potential reservoirs for the transference of antimicrobial resistance to humans. Indeed, a recent study demonstrated acquired resistance to azithromycin, spectinomycin, enrofloxacin, and lincomycin in some feline *H. heilmannii* isolates [29]. Thus, veterinary clinicians should be prudent when establishing a therapeutic protocol based solely on the presence of these bacteria in the feline stomach. Two important consequences can arise from this clinical action: interference with the gastric microbiome and homeostasis and contribution to the increase and perpetuation of antibiotic resistance, which remains a serious and emergent concern in both human and veterinary medicine.

## 5. Conclusions

In this study, the presence of *Helicobacter* spp. was assessed with four methods, and an overall occurrence of 74.6% was reported, with *H. heilmannii* being the most frequently identified species. Mixed colonization was also detected in 67.9% of the cats, demonstrating the richness and diversity of the bacterial combinations that can be found in the feline stomach. 

Our results reflect a soft relationship between bacteria belonging to the genus *Helicobacter* and the feline stomach, reinforcing the high degree of adaptation of these bacteria to this host. We hypothesize that *Helicobacter* spp. might become pathogenic under specific conditions that might lead to the deregulation or imbalance of the host complex gastric microenvironment. However, further studies are needed to validate this hypothesis.

## Figures and Tables

**Table 1:** Gastric samples subjected to evaluation with different methods.

Cats (*n* = 71)	HE	MG	IHC	PCR
Body + Antrum	16	16	16	16
Body	45	45	45	50
Antrum	5	5	5	5
Total number of samples evaluated	66	66	66	71

Legend: HE—hematoxylin–eosin; MG—modified Giemsa stain; IHC—immunohistochemistry; PCR—polymerase chain reaction.

**Table 2:** Results of histopathological evaluation of feline gastric samples.

**Histopathological Features (Day et al., 2008)**		**Frequency (Percentage and Number)**	
**Normal**		**30.3 (20/66)**	
	Without bacteria		*35.0 (7/20)*
	With bacteria		*65.0 (13/20)*
**Inflammation**		**69.7 (46/66)**	
	Mild gastritis		*43.5 (20/46)*
	Moderate gastritis		*45.6 (21/46)*
	Marked gastritis		*10.9 (5/46)*
Intraepithelial lymphocytes *		**48.4 (30/62)**	
	Mild		*33.3 (10/30)*
	Moderate		*26.7 (8/30)*
	Marked		*40.0 (12/30)*
**Fibrosis or mucosal atrophy**		**34.8 (23/66)**	
	Mild		*52.2 (12/23)*
	Moderate		*47.8 (11/23)*
**Neutrophilic infiltration**		**12.1 (8/66)**	

* This parameter was not possible to evaluate in 4 animals due artefactual constraints that resulted in a lack of proper epithelial representation.

**Table 3:** Detection of *Helicobacter* spp. in the different compartments of the feline stomach according to different diagnostic methods.

Gastric Region	*Helicobacter* spp.-Positive Results Obtained with Different Methods % (Number of Positive Results/Total Number of Cases)				
	**HE**	**MG**	**IHC**	**PCR**	**Regardless of Method**
Overall Stomach	37.9% (25/66)	48.5% (32/66)	71.2% (47/66)	39.4% (28/71)	74.6% (53/71)
Body (*n* = 61)	34.4% (21/61)	44.3% (27/61)	70.5% (43/61)	45.9% (28/61)	
Antrum (*n* = 21)	47.6% (10/21)	76.2% (16/21)	71.4% (15/21)	23.8% (5/21)	

Legend: HE—hematoxylin–eosin; MG—modified Giemsa stain; IHC—immunohistochemistry; PCR—polymerase chain reaction.

**Table 4:** Specific *Helicobacter* species detected by PCR in different compartments of the feline stomach.

Specific PCR-*Helicobacter* spp. Positive Results	Gastric Region % (nr/Total)		
	Overall Stomach *	Body (*n* = 28)	Antrum (*n* = 5)
*H. heilmannii*	71.4 (20/28)	10.7 (3/28)	
*H. felis*	46.4 (13/28)	3.6 (1/28)	
*H. bizzozeronnii*	39.3 (11/28)	7.1 (2/28)	
*H. salomonis*	50.0 (14/28)	7.1 (2/28)	
*H. heilmannii-like*	3.6 (1/28)	3.6 (1/28)	
*H. heilmannii + H. felis*	39.3 (11/28)	10.7 (3/28)	
*H. heilmannii + H. felis + H. bizzozeronnii*	28.6 (8/28)	10.7 (3/28)	
*H. heilmannii + H. felis + H. bizzozeronnii + H. salomonis*	57.1 (16/28)	10.7 (3/28)	40.0 (2/5)
*H. heilmannii + H. felis + H. salomonis*	35.7 (10/28)	7.1 (2/28)	20.0 (1/5)
*H. heilmannii + H. bizzozeronnii*	25 (7/28)	3.6 (1/28)	
*H. heilmannii + H. salomonis*	35.7 (10/28)	17.9 (5/28)	40.0 (2/5)
*H. felis + H. bizzozeronnii + H. salomonis + H. heilmannii-like*	46.4 (13/28)	3.6 (1/28)	
*H. bizzozeronnii + H. salomonis*	17.9 (5/28)	3.6 (1/28)	

* Regardless of gastric region.

**Table 5:** Association between gastric histopathological alterations and the presence of *Helicobacter* spp. in cats.

		Frequency (Percentage and Number)	*p* *
**Gastritis**			
	Normal	65% (13/20)	0.408
	Abnormal	78.3% (36/46)	
**Surface epithelial injury**			
	Normal	75% (3/4)	0.985
	Abnormal	74.6% (44/59)	
**Gastric pit epithelial injury**			
	Normal	70.0% (20/29)	0.342
	Abnormal	79.4% (27/34)	
**Fibrosis/mucosal atrophy**			
	Normal	69.8% (30/43)	0.256
	Abnormal	82.6% (19/23)	
**Intraepithelial lymphocytes**			
	Normal	62.5% (20/32)	**0.030**
	Abnormal	86.7% (26/30)	
***Lamina propria* lymphocytes and plasma cells**			
	Normal	65% (13/20)	0.258
	Abnormal	78.3% (36/46)	
***Lamina propria* eosinophils**			
	Normal	72.9% (43/59)	0.463
	Abnormal	85.7% (6/7)	
***Lamina propria* neutrophils**			
	Normal	74.1% (43/58)	0.958
	Abnormal	75% (6/8)	
**Other inflammatory cells**			
	Normal	75.4% (46/61)	0.449
	Abnormal	60% (3/5)	
**Lymphofollicular hyperplasia**			
	Normal	75% (45/60)	0.656
	Abnormal	66.7% (4/6)	

* Differences were considered statistically significant at *p*-values of <0.05.

## Data Availability

Not applicable.

## Supplementary Materials

The following supporting information can be downloaded at: https://www.mdpi.com/article/10.3390/vetsci9050228/s1, Table S1: Primers sequences used for conventional PCR and thermo cycling conditions; Table S2: Table summarizing the histopathological alterations and colonization density observed in the feline stomach, according to the positive NHPH species-specific PCR results, final *Helicobacter* positive and negative results regardless of the method used.
