# Supplementary material for: Helicobacter spp. in the Stomach of Cats: Successful Colonization and Absence of Relevant Histopathological Alterations Reveals High Adaptation to the Host Gastric Niche
Source: Vet Sci. 2022 May 10;9(5):228. doi: 10.3390/vetsci9050228 (PMC9148067; doi:10.3390/vetsci9050228)
Supplement: Supplementary file 1 [file vetsci-09-00228-s001.zip › vetsci-1678325-supplementary.pdf]

veterinary sciences

MDPI

Table S1. Primers sequences used for conventional PCR and thermo cycling conditions.

Helicobacter species	Primer	Nucleotide sequence	Target gene	Amplicon size (bp)	Thermal cycle conditions			Positive control
					Nr. Cycles	Temp. (C)	Time	
Helicobacter spp.	Hcom1	GTA AAG GCT CAC CAA GGC TAT	16S	389	40	94	1'	*
						63	1'	
	Hcom2	CCA CCT ACC TCT CCC ACA CTC	16S			72	1'	
H. pylori	BFHpyl_F1	AAA GAG CGT GGT TTT CAT GGC G	ureAB	217	45	94	30''	26695
						59	30''	
	BFHpyl_R1	GGG TTT TAC CGC CAC CGA ATT TAA	ureAB			72	1'	
H. heilmannii	Hh-IceA-FWQ	GGC TCT GCG TAG GAC CTG CTA CAG AAG CTC TC	ureAB	135	30	94	30''	ASB1.4
						55	30''	
	Hh-IceA-RVQ	GGC TGT AGG GAT TTG TTG AGG AGA AAT G	ureAB			72	30''	
H. felis	BFHfelis_F2	GCT GGT GGC ATC GAT ACG CAT	ureAB	154	45	94	30''	CS1
						60	30''	
	BFHfelis_R2	TTT TTA GAT TAG CGC GTC CGG GA	ureAB			72	1'	
H. bizzozeronnii	Hbizz_FQ_PAR	CGC TTT GAA CCC GGT GAG AAA A	ure A	172	45	94	1'	R1053
						58	1'	
	Hbizz_RQ_PAR	TAT CGC AAC CGC AAT TCA CAA CA	ure A			72	1'	
H. salomonis	Hsal_FQ_PAR	CTC TTA TGA GTT GGA CTT GGT GCT CAC CAA T	ure AB	91	45	94	30''	R1051
						61	30''	
	Hsal_RQ_PAR	TTT GCC ATC TTT AAT TCC AAT GTC GGC	ure AB			72	1'	
H. ailurogastricus	Ha-Lpsa-FWQ	CTT GAG TAC GGC GAT GTC AAT	Lpsa	136	30	94	30''	ASB7.1
						55	30''	
	Ha-Lpsa-RVQ	GGG GAA AAA TGT GCT TGA AGT	Lpsa			72	30''	

* One of the *Helicobacter* species extracted from pure cultures was used as positive control.

Table S2. Table summarizing the histopathological alterations and colonization density observed in the feline stomach, according to the positive NHPH species-specific PCR results, final *Helicobacter* positive and negative results regardless of the method used.

	PCR positive results with specific species identification, regardless gastric location (n=28)													Final positive result regardless the method used % (nr/total) (n=53)*	Final negative result regardless the method used % (nr/total) (n=18)*
	Hh	***Hf***	***Hb***	***Hs***	***Hh-l***	***Hh+ Hf***	***Hh+Hf+ Hb***	***Hh+Hf+ Hb+Hs***	***Hh+Hf+Hs***	***Hh+Hb***	***Hh+Hs***	***Hf+Hb+ Hs+Hh-l***	***Hb+Hs***		
Histopathology grading (Day *et al*., 2008)															
Normal	3.6 (1/28)	3.6 (1/28)	0	3.6 (1/28)	0	3.6 (1/28)	7.1 (2/28)	7.1 (2/28)	3.6 (1/28)	0	3.6 (1/28)	3.6 (1/28)	0	24.5 (13/53)	38.9 (7/18)
Mild gastritis	3.6 (1/28)	0	3.6 (1/28)	0	3.6 (1/28)	3.6 (1/28)	0	3.6 (1/28)	3.6 (1/28)	0	3.6 (1/28)	0	3.6 (1/28)	22.6 (12/53)	44.4 (8/18)
Moderate gastritis	0	0	0	0	0	3.6 (1/28)	3.6 (1/28)	0	0	3.6 (1/28)	7.1 (2/28)	0	0	35.9 (19/53)	11.1 (2/18)
Marked gastritis	0	0	0	3.6 (1/28)	0	0	0	0	0	0	0	0	0	9.4 (5/53)	0
ID	3.6 (1/28)	0	3.6 (1/28)	0	0	0	0	0	0	0	3.6 (1/28)	0	0	7.6 (4/53)	5.6 (1/18)
Surface epithelial injury															
1 - Normal	0	0	0	0	0	0	0	3.6 (1/28)	0	0	0	0	0	5.7 (3/53)	5.6 (1/18)
2 - Mild	7.1 (2/28)	3.6 (1/28)	3.6 (1/28)	7.1 (2/28)	3.6 (1/28)	7.1 (2/28)	7.1 (2/28)	7.1 (2/28)	3.6 (1/28)	3.6 (1/28)	10.7 (3/28)	0	3.6 (1/28)	69.8 (37/53)	77.8 (14/18)
3 - Moderate	0	0	0	0	0	3.6 (1/28)	0	0	3.6 (1/28)	0	3.6 (1/28)	0	0	9.4 (5/53)	0
4 - Marked	0	0	0	0	0	0	0	0	0	0	0	0	0	3.8 (2/53)	5.6 (1/18)
ID	3.6 (1/28)	0	3.6 (1/28)	0	0	0	3.6 (1/28)	0	0	0	3.6 (1/28)	3.6 (1/28)	0	11.3 (6/53)	11.1 (2/18)
Gastric pit epithelial injury															
1 - Normal	7.1 (2/28)	3.6 (1/28)	0	7.1 (2/28)	3.6 (1/28)	0	3.6 (1/28)	3.6 (1/28)	3.6 (1/28)	0	10.7 (3/28)	0	3.6 (1/28)	37.7 (20/53)	50.0 (9/18)
2 - Mild	0	0	3.6 (1/28)	0	0	7.1 (2/28)	3.6 (1/28)	7.1 (2/28)	0	3.6 (1/28)	3.6 (1/28)	0	0	37.7 (20/53)	27.8 (5/18)
3 - Moderate	0	0	0	0	0	3.6 (1/28)	0	0	3.6 (1/28)	0	0	0	0	11.3 (6/53)	5.6 (1/18)
4 - Marked	0	0	0	0	0	0	0	0	0	0	0	0	0	1.9 (1/53)	5.6 (1/18)
ID	3.6 (1/28)	0	3.6 (1/28)	0	0	0	3.6 (1/28)	0	0	0	3.6 (1/28)	3.6 (1/28)	0	11.3 (6/53)	11.1 (2/18)

Fibrosis/ mucosal atrophy															
1 - Normal	7.1 (2/28)	3.6 (1/28)	3.6 (1/28)	7.1 (2/28)	0	7.1 (2/28)	10.7 (3/28)	10.7 (3/28)	7.1 (2/28)	3.6 (1/28)	14.3 (4/28)	3.6 (1/28)	3.6 (1/28)	56.6 (30/53)	72.2 (13/18)
2 - Mild	0	0	0	0	0	3.6 (1/28)	0	0	0	0	0	0	0	17.0 (9/53)	16.7 (3/18)
3 - Moderate	0	0	0	0	3.6 (1/28)	0	0	0	0	0	0	0	0	18.9 (10/53)	5.6 (1/18)
4 - Marked	0	0	0	0	0	0	0	0	0	0	0	0	0	0	0
ID	3.6 (1/28)	0	3.6 (1/28)	0	0	0	0	0	0	0	3.6 (1/28)	0	0	7.5 (4/53)	5.6 (1/18)
Intraepithelial lymphocytes															
1 - Normal	7.1 (2/28)	3.6 (1/28)	0	3.6 (1/28)	0	3.6 (1/28)	0	3.6 (1/28)	3.6 (1/28)	0	3.6 (1/28)	3.6 (1/28)	0	37.7 (20/53)	66.7 (12/18)
2 - Mild	0	0	0	0	0	0	3.6 (1/28)	0	3.6 (1/28)	3.6 (1/28)	7.1 (2/28)	0	3.6 (1/28)	15.1 (8/53)	11.1 (2/18)
3 - Moderate	0	0	0	3.6 (1/28)	0	3.6 (1/28)	0	3.6 (1/28)	0	0	3.6 (1/28)	0	0	13.2 (7/53)	5.6 (1/18)
4 - Marked	0	0	3.6 (1/28)	0	0	0	3.6 (1/28)	3.6 (1/28)	0	0	0	0	0	20.8 (11/53)	5.6 (1/18)
ID	3.6 (1/28)	0	3.6 (1/28)	0	3.6 (1/28)	3.6 (1/28)	3.6 (1/28)	0	0	0	3.6 (1/28)	0	0	13.2 (7/53)	11.1 (2/18)
Lamina propria lymphocytes and plasma cells															
1 - Normal	3.6 (1/28)	3.6 (1/28)	0	3.6 (1/28)	0	3.6 (1/28)	7.1 (2/28)	7.1 (2/28)	3.6 (1/28)	0	3.6 (1/28)	3.6 (1/28)	0	24.5 (13/53)	38.9 (7/18)
2 - Mild	3.6 (1/28)	0	3.6 (1/28)	0	3.6 (1/28)	3.6 (1/28)	0	3.6 (1/28)	3.6 (1/28)	0	3.6 (1/28)	0	3.6 (1/28)	22.6 (12/53)	44.4 (8/18)
3 - Moderate	0	0	0	0	0	3.6 (1/28)	3.6 (1/28)	0	0	3.6 (1/28)	7.1 (2/28)	0	0	35.8 (19/53)	11.1 (2/18)
4 - Marked	0	0	0	3.6 (1/28)	0	0	0	0	0	0	0	0	0	9.4 (5/53)	0
ID	3.6 (1/28)	0	3.6 (1/28)	0	0	0	0	0	0	0	3.6 (1/28)	0	0	7.5 (4/53)	5.6 (1/18)
Lamina propria eosinophils															
1 - Normal	7.1 (2/28)	3.6 (1/28)	3.6 (1/28)	7.1 (2/28)	3.6 (1/28)	10.7 (3/28)	10.7 (3/28)	10.7 (3/28)	7.1 (2/28)	3.6 (1/28)	14.3 (4/28)	3.6 (1/28)	3.6 (1/28)	81.1 (43/53)	88.9 (16/18)
2 - Mild	0	0	0	0	0	0	0	0	0	0	0	0	0	5.7 (3/53)	5.6 (1/18)
3 - Moderate	0	0	0	0	0	0	0	0	0	0	0	0	0	3.8 (2/53)	0
4 - Marked	0	0	0	0	0	0	0	0	0	0	0	0	0	1.9 (1/53)	0
ID	3.6 (1/28)	0	3.6 (1/28)	0	0	0	0	0	0	0	3.6 (1/28)	0	0	7.5 (4/53)	5.6 (1/18)
Lamina própria neutrophils															

1 - Normal	7.1 (2/28)	3.6 (1/28)	3.6 (1/28)	7.1 (2/28)	3.6 (1/28)	10.7 (3/28)	10.7 (3/28)	10.7 (3/28)	7.1 (2/28)	3.6 (1/28)	14.3 (4/28)	3.6 (1/28)	3.6 (1/28)	81.1 (43/53)	83.3 (15/18)
2 - Mild	0	0	0	0	0	0	0	0	0	0	0	0	0	7.5 (4/53)	5.6 (1/18)
3 - Moderate	0	0	0	0	0	0	0	0	0	0	0	0	0	1.9 (1/53)	5.6 (1/18)
4 - Marked	0	0	0	0	0	0	0	0	0	0	0	0	0	1.9 (1/53)	0
ID	3.6 (1/28)	0	3.6 (1/28)	0	0	0	0	0	0	0	3.6 (1/28)	0	0	7.5 (4/53)	5.6 (1/18)
Other inflammatory cells															
1 - Normal	7.1 (2/28)	3.6 (1/28)	3.6 (1/28)	7.1 (2/28)	3.6 (1/28)	10.7 (3/28)	10.7 (3/28)	10.7 (3/28)	7.1 (2/28)	3.6 (1/28)	14.3 (4/28)	3.6 (1/28)	3.6 (1/28)	86.8 (46/53)	83.3 (15/18)
2 - Mild	0	0	0	0	0	0	0	0	0	0	0	0	0	5.7 (3/53)	11.1 (2/18)
3- Moderate	0	0	0	0	0	0	0	0	0	0	0	0	0	0	0
4 - Marked	0	0	0	0	0	0	0	0	0	0	0	0	0	0	0
ID	3.6 (1/28)	0	3.6 (1/28)	0	0	0	0	0	0	0	3.6 (1/28)	0	0	7.5 (4/53)	5.6 (1/18)
Lymphofollicular hyperplasia															
1 - Normal	7.1 (2/28)	3.6 (1/28)	3.6 (1/28)	7.1 (2/28)	3.6 (1/28)	10.7 (3/28)	10.7 (3/28)	10.7 (3/28)	7.1 (2/28)	3.6 (1/28)	14.3 (4/28)	3.6 (1/28)	3.6 (1/28)	84.9 (45/53)	83.3 (15/18)
2 - Mild	0	0	0	0	0	0	0	0	0	0	0	0	0	3.8 (2/53)	0
3 - Moderate	0	0	0	0	0	0	0	0	0	0	0	0	0	3.8 (2/53)	5.6 (1/18)
4 - Marked	0	0	0	0	0	0	0	0	0	0	0	0	0	0	5.6 (1/18)
ID	3.6 (1/28)	0	3.6 (1/28)	0	0	0	0	0	0	0	3.6 (1/28)	0	0	7.5 (4/53)	5.6 (1/18)
Bacterial density (based on IHC results)															NA
0	0	3.6 (1/28)	0	0	0	0	0	0	0	0	0	0	0	7.5 (4/53)	
+	0	0	0	0	3.6 (1/28)	0	0	0	0	0	0	0	0	3.8 (2/53)	
++	3.6 (1/28)	0	0	0	0	7.1 (2/28)	3.6 (1/28)	0	0	0	0	0	0	11.3 (6/53)	
+++	3.6 (1/28)	0	3.6 (1/28)	7.1 (2/28)	0	3.6 (1/28)	7.1 (2/28)	10.7 (3/28)	7.1 (2/28)	3.6 (1/28)	14.3 (4/28)	3.6 (1/28)	3.6 (1/28)	69.8 (37/53)	
ID	3.6 (1/28)	0	3.6 (1/28)	0	0	0	0	0	0	0	3.6 (1/28)	0	0	7.5 (4/53)	5.6 (1/18)

Legend: Hh, *H.heilmannii*; Hf, *H. felis*; Hb, *H. bizzozeronnii*; Hs, *H. salomonis*; Hh-l, *H. heilmannii-like*. ID, Indeterminate; NA, not applicable.

* It was not possible to evaluate histologically 5 out of 71 animals due to the small amount of material present
